# Why we should monitor disparities in old-age mortality with the modal age at death

**DOI:** 10.1371/journal.pone.0263626

**Published:** 2022-02-09

**Authors:** Viorela Diaconu, Alyson van Raalte, Pekka Martikainen

**Affiliations:** 1 Lifespan Inequalities Research Group, Max Planck Institute for Demographic Research, Rostock, Germany; 2 Population Research Unit (PRU), Faculty of Social Sciences, University of Helsinki, Helsinki, Finland; 3 Centre for Health Equity Studies (CHESS), Department of Public Health Sciences, Stockholm University and Karolinska Institutet, Stockholm, Sweden; Universidade Federal de Minas Gerais, BRAZIL

## Abstract

Indicators based a fixed “old” age threshold have been widely used for assessing socioeconomic disparities in mortality at older ages. Interpretation of long-term trends and determinants of these indicators is challenging because mortality above a fixed age that in the past would have reflected old age deaths is today mixing premature and old-age mortality. We propose the modal (i.e., most frequent) age at death, *M*, an indicator increasingly recognized in aging research, but which has been infrequently used for monitoring mortality disparities at older ages. We use mortality and population exposure data by occupational class over the 1971-2017 period from Finnish register data. The modal age and life expectancy indicators are estimated from mortality rates smoothed with penalized *B*-splines. Over the 1971-2017 period, occupational class disparities in life expectancy at 65 and 75 widened while disparities in *M* remained relatively stable. The proportion of the group surviving to the modal age was constant across time and occupational class. In contrast, the proportion surviving to age 65 and 75 has roughly doubled since 1971 and showed strong occupational class differences. Increasing socioeconomic disparities in mortality based on fixed old age thresholds may be a feature of changing selection dynamics in a context of overall declining mortality. Unlike life expectancy at a selected fixed old age, *M* compares individuals with similar survival chances over time and across occupational classes. This property makes trends and differentials in *M* easier to interpret in countries where old-age survival has improved significantly.

## Introduction

Socioeconomic inequalities in mortality persist into old age and have widened over time [1–8]. As the lion’s share of mortality currently occurs at older ages, there has been a growing interest in past years in analyzing socioeconomic inequalities in mortality at these ages. Old-age mortality disparities are usually assessed using traditional indicators of mortality which are based on a fixed “old” age threshold, most often age 65; for example, life expectancy at age 65 or age-standardized death rates above age 65. Interpretation of the trends and determinants of these indicators may be challenging because of the substantial decline in the proportion of deaths occurring before these ages and the postponement of mortality to increasingly higher ages over time. In other words, mortality above a fixed age threshold that in the past would have reflected old age deaths, is today mixing premature and old age mortality. There is therefore a growing need to assess social disparities in mortality with measures that are free from an arbitrary selection of the “old” age threshold.

We propose the adult modal age at death, *M*, that is the age (beyond infancy) at which the largest single number of deaths occur, to be used as an indicator for the analyses of mortality disparities at older ages. Under a given mortality regime, *M* represents the most common or “typical” length of life among adults. Compared to conventional measures of old-age mortality such as life expectancy, *M* has the following desirable properties: (1) freedom from an arbitrary selection of the “old”’ age threshold, (2) solely determined by mortality at older ages [9, 10].

Over the last two decades, *M* has been used for monitoring trends in old-age survival improvements, all social classes combined, in a number of low mortality countries [9, 11–18]. Despite its intuitive meaning and desirable properties, *M* has not been commonly used for examining socioeconomic differences in mortality. To date, a few studies have used *M* for assessing mortality disparities at older ages, but none have captured long-time trends [11, 19].

The objective of this paper is twofold. First, it aims to highlight the advantages of *M* for monitoring socioeconomic differences in mortality at older ages. Second, it compares long-time trends in *M* with the more widely-used life expectancy at age 65 and 75, *e*_65_, *e*_75_. We focus on the special case of Finland as it is one of the few countries with exceptionally high-quality mortality register data covering the entire population for nearly 50 years.

## Data and methods

### Data

The data consists of observed death counts and population exposures by single year of age (31 and above), sex, and occupational class covering the period 1971–2017 in Finland. Data by sex, occupational class, calendar year, and single year of age was not available for ages younger than 31 in this study setting. The mortality and population exposure series by socioeconomic status were obtained by linking the individual-level census and population register data of all Finns to death records using personal identification codes. The data were collected for routine administrative registration purposes and, therefore, informed consent of the participants was not obtained. These register data can be used for scientific purposes under the Personal Data Act and the Statistics Act. Statistics Finland anonymised the data prior to providing them to researchers. The study has been approved by Statistics Finland Board of Ethics (permit TK-53–339- 13).

We used occupation-based social class as our socioeconomic indicator which was measured at the time of each census and updated every fifth year. Women and men were allocated into the following classes based on their current or previous occupation: 1. upper non-manual (e.g. doctors and teachers), 2. lower non-manual (e.g. shop salespersons and nurses), 3. manual workers (e.g. construction workers, bus drivers, and cleaners), and 4. others (e.g. farm and forestry workers, students, entrepreneurs, unknown occupational class). We focus on mortality differences between the three first classes only given the substantial heterogeneity and large compositional changes that have occurred over time within the fourth group. For retired and unemployed individuals as well as for those with unknown occupation at the time of the census, information was gathered from earlier censuses. Those whose main activity was household work were classified according to the occupation of the head of the household. Immigrants were removed from the data set since there was no information on their previous occupational class outside Finland and emigrants were censored at emigration. S1 Fig illustrates changes over time in the distribution of these occupational classes for males and females.

### Methods

We monitor changes in mortality across time and occupational class using the modal age at death *M* and life expectancies conditional on survival to older ages (i.e., 65, 75), *e*_*x*_. For ease of graphical comparison with *M*, the *e*_*x*_ refer to the average attained age at death as opposed to the average remaining life expectancy.

Death counts at older ages are often “noisy”, i.e. fluctuating from one age to the next, making it challenging to estimate the modal age precisely. Without precision, increases or decreases in *M* from changing mortality levels could not be easily distinguished from statistical fluctuation. To overcome this, we smooth mortality rates in two dimensions (i.e., over age and calendar year) with penalized *B*-splines (*P*-splines) [20, 21]. *B*-splines provide flexibility and an accurate fit of the data, while the penalty, acting on the coefficients of adjacent *B*-splines, ensures that the resulting fit is smooth. The compromise between smoothness and precision of fit to the observed data is controlled by a smoothing parameter included in the model. The smoothing parameter is selected using the Bayesian Information Criterion (BIC) [22], as this criterion has been proven the most suitable in the context of mortality data [21]. Smoothing was done separately for each sex and occupational class, with a unique smoothing parameter for each strata, which ensures an accurate fit to and a smooth replication of the observed data.

The P-spline method has been proven highly effective for smoothing mortality rates and hence for obtaining smooth forces of mortality [20, 21, 23].

**Fig 1:**
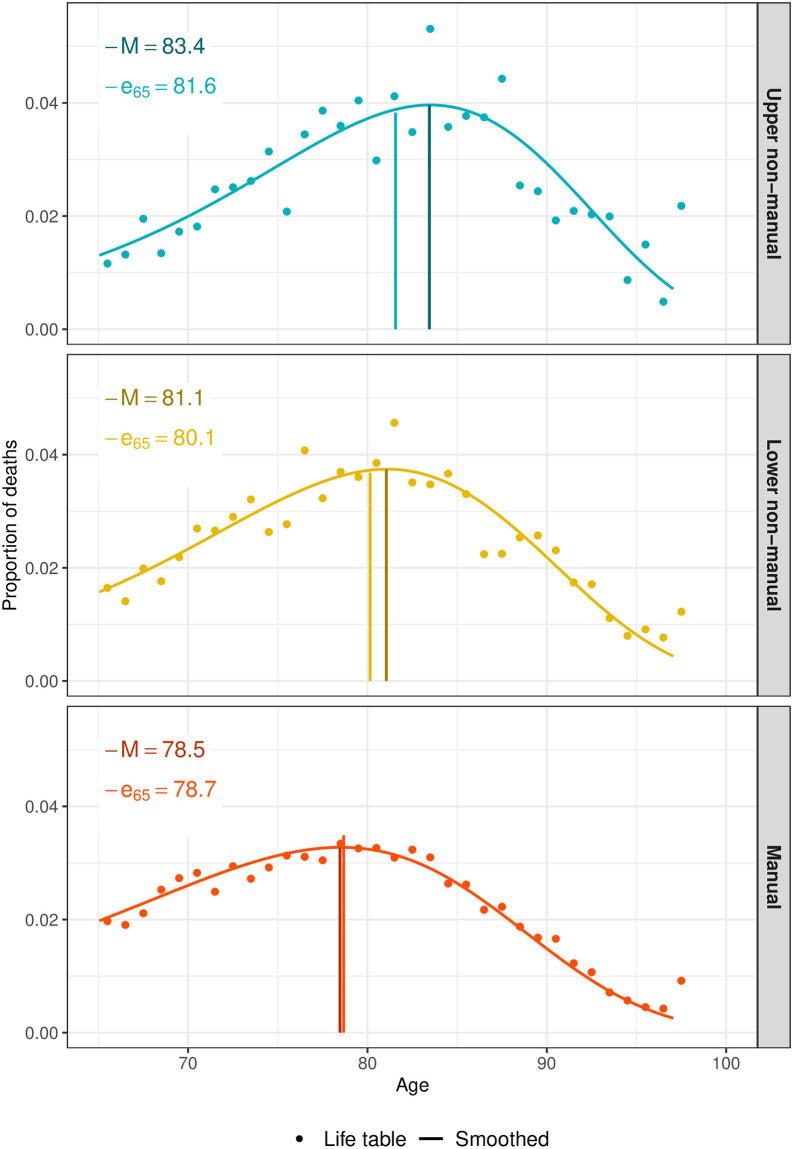
Life table deaths and two-dimension *P*-spline smoothed deaths with corresponding modal age at death, *M*, and conditional life expectancy at age 65, by occupational class, Finnish males, 1994. Smoothing was based on ages above 31.

Smoothing was done separately for each sex and occupational class. The *P*-spline smooth age-at- death distributions are in fact a smooth representation of the life table distribution by age Fig 1. More details on the *P*-spline smoothing method can be found in the Appendix of [17].

*M* and *e*_*x*_ are obtained with great numerical precision from the same occupation- and sex-specific *P*-spline smooth density function using standard numerical techniques (Eq A.2 and A.3 in S1 Appendix). As shown in Fig 1, the occupation- and sex-specific *P*-spline smooth density function is the smooth representation of the life table distribution of deaths by age. Deriving *M* and *e*_*x*_ from the smooth age-at-death distribution is based on the same principle as deriving *M* and *e*_*x*_ from the life table age-at-death distribution, with the exception that the former is in a continuous setting while the later is in a discrete setting.

## Results

### *M* for measuring socioeconomic inequalities in mortality

**Fig 2:**
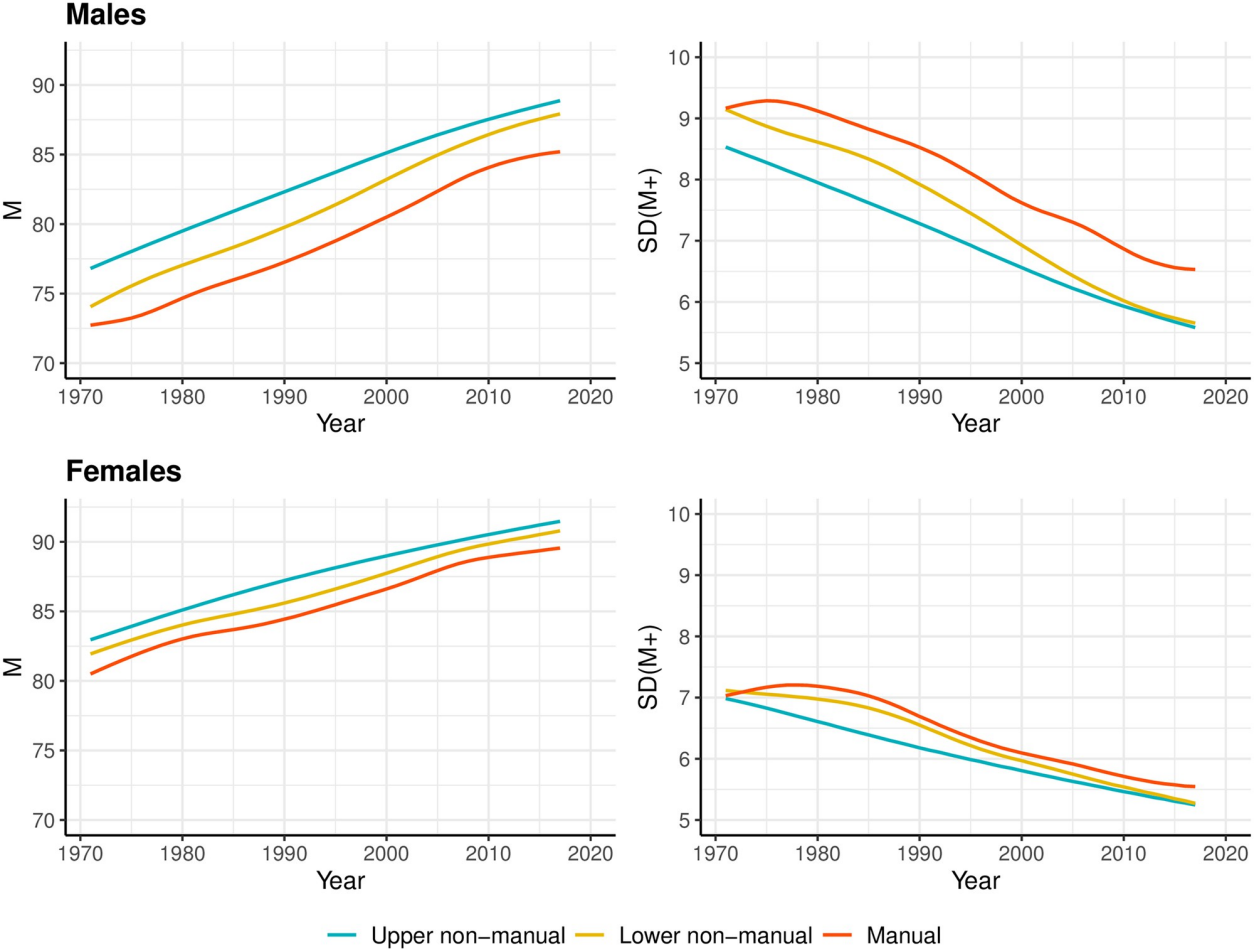
Trends in modal age at death, *M* and standard deviation above the mode, *SD(M+)*, by occupational class and sex, Finland, 1971–2017. Smoothing was based on ages above 31.

Fig 2 illustrates trends in modal age at death, *M*, and in standard deviation above the mode, *SD*(*M*+), by occupational class and sex in Finland over the 1971–2017 period. As shown, increases in *M* were accompanied by a decline in *SD*(*M*+) for each sex and occupational class. This compression of mortality at older ages was greater for the lower non-manual classes compared to the other two occupational classes, as shown by a faster increase in *M* and a more rapid decline in *SD*(*M*+) over the study period. Towards the end of the follow-up period the lower non-manual class converges with the upper non-manual class.

Occupational inequalities in length of life as well as variability of age at death above the mode remained quite stable between the upper non-manual and manual classes, and were always much larger for men than women. Men also had higher *SD*(*M*+) compared to women overall, but also experienced stronger old-age mortality compression over this period.

### Comparison of *M* with more widely-used indicators of old-age mortality

**Fig 3:**
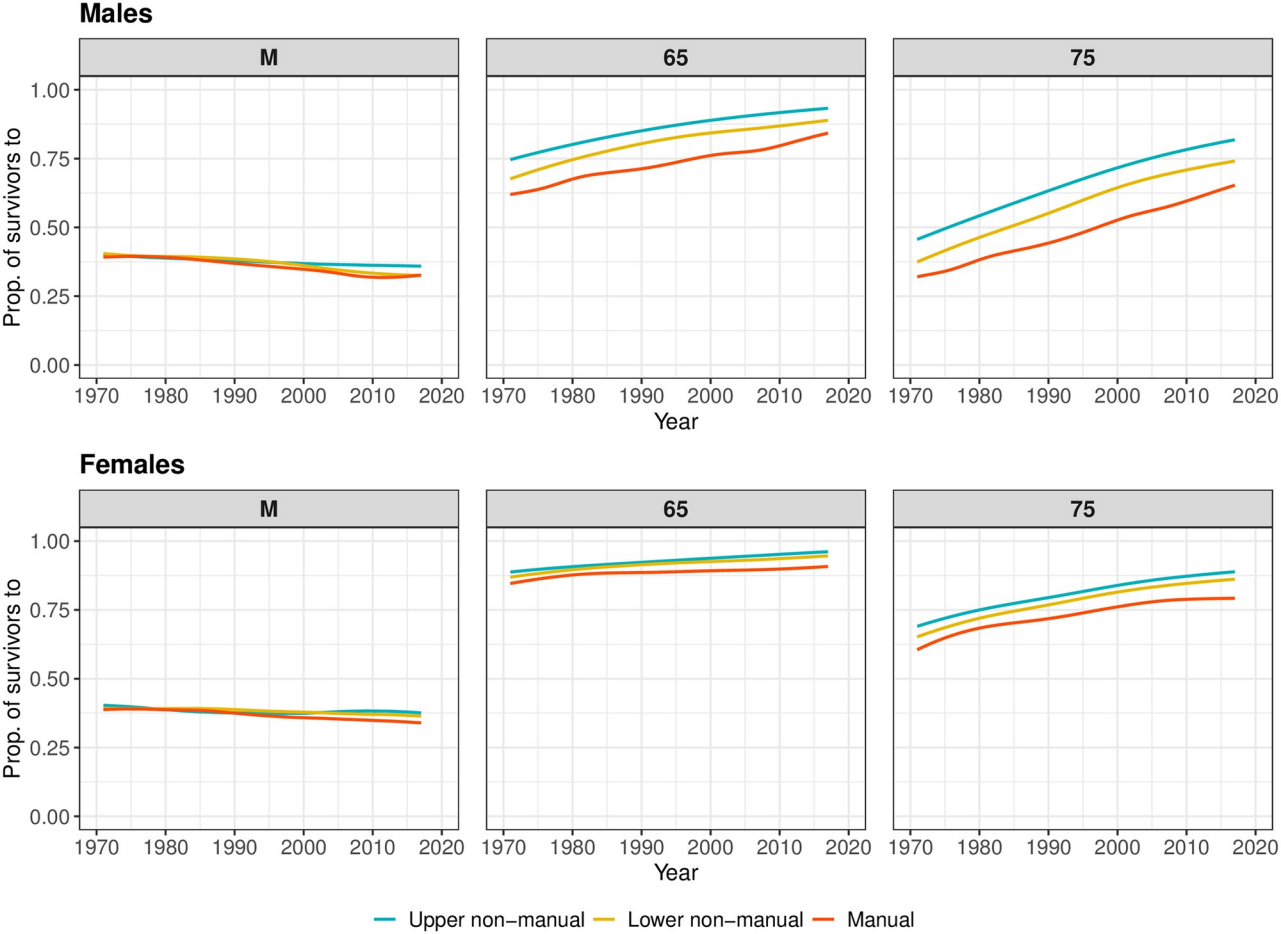
Proportion of 31 years olds surviving to *M*, 65, and 75, based on smoothed period mortality data, by occupational class and sex, Finland, 1971–2017. Smoothing was based on ages above 31.

Fig 3 shows trends in the proportion of 31-year olds surviving to the occupation-specific mode *M*, and to ages 65 and 75 by occupational class and sex over the 1971–2017 period in Finland, based on the smoothed period life tables. For both males and females, the proportion surviving to *M* remained relatively constant over time for all occupational classes at just below 40%. In contrast, the proportion surviving to age 65 and 75 followed an upward trend throughout the 1971–2017 period, for both sexes and for each occupational class. The most substantial increase is observed for survival to age 75, particularly for males. For instance, about 40% of males in lower non-manual occupations reached age 75 in 1971. By 2017, this figure was around 75%. An increase of such magnitude is also observed for the other two occupational classes. Also noteworthy is that there were virtually no occupational class differences in the proportion surviving to *M*, but there was on average 10 to 20 percentage point difference between upper non-manual and manual classes in surviving to age 65 and 75 over the observation period.

**Fig 4:**
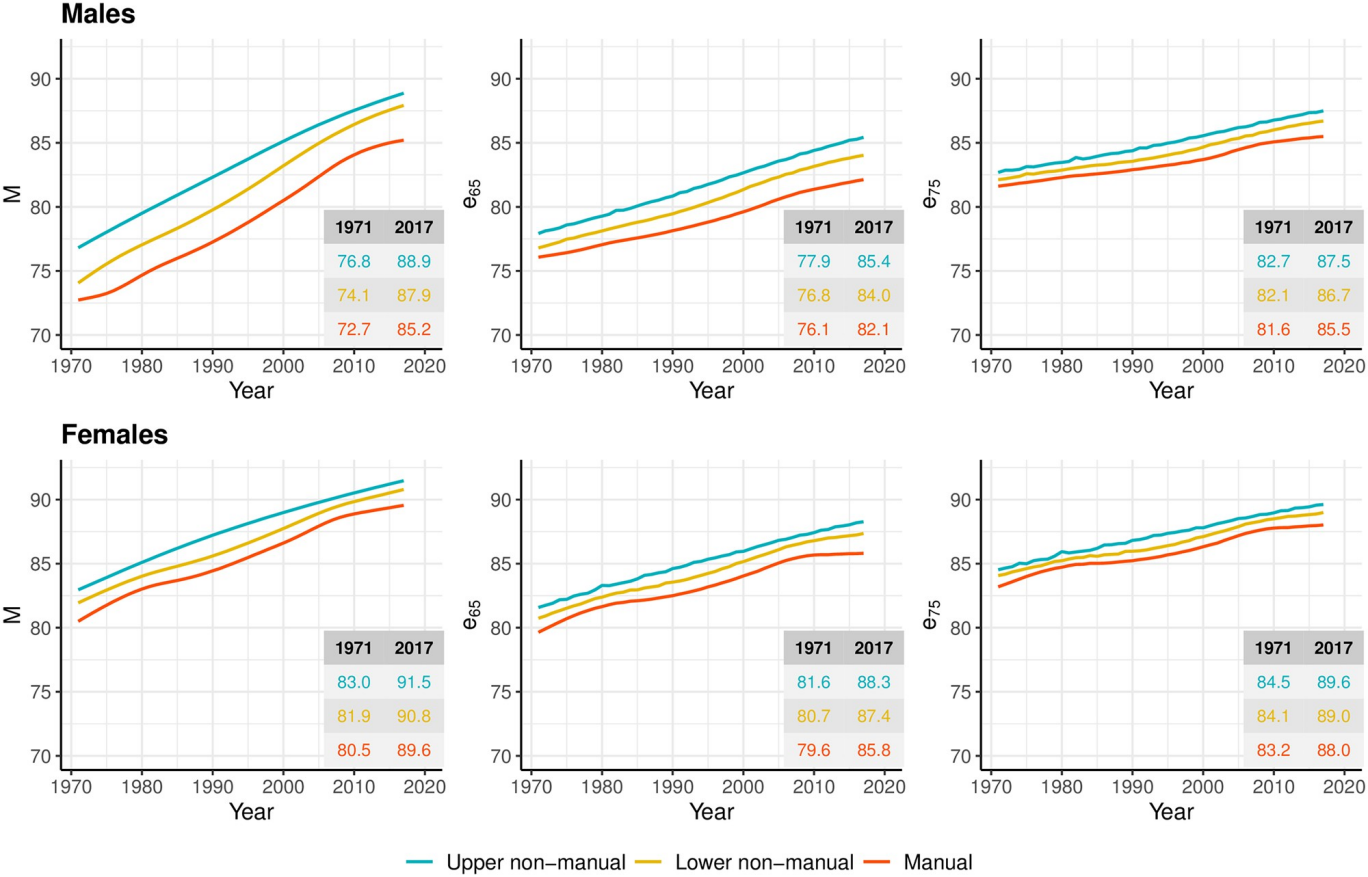
Modal age at death (*M*) and conditional life expectancies at age 65 and 75 (e_65_, e_75_) based on smoothed age-at-death distributions by occupational class and sex, Finland, 1971–2017. Smoothing was based on ages above 31.

Fig 4 illustrates trends in *M*, *e*_65_, and *e*_75_ by occupational class and sex in Finland over the 1971–2017 period. An occupational class gradient in mortality at older ages is observed regardless of the indicator used. However, the magnitude of the gradient depends on the indicator used and varies by calendar year and sex. For instance, in the early 1970s, *M* for males in upper non-manual classes is about 4 years higher than in manual classes. The difference in *e*_*x*_ between these two classes is 2 years at age 65 and 1 year at age 75. Compared to males, occupational class differences among females are smaller regardless of the indicator used. In 1971, females in upper non-manual occupations had a *M* about 2 years higher than their counterparts in manual occupations. The difference between these two classes in e65 and e75 amounts to 1.8 and 1.3 years respectively.

The indices follow an upward trend for all occupational classes and for both sexes over the 1971–2017 period. However, *M* increases at a faster pace compared to *e*_65_ and *e*_75_, especially in lower occupational classes. Compared to males, gains in *M* for females are more modest. In contrast, the *e*_*x*_ indicators exhibit larger gains in the higher than lower occupational classes.

As a result, the occupational class gradient at older ages follows different trends depending on whether inequalities are assessed with *M* or *e*_*x*_ indicators. Among males, inequalities in *M* were stable from 1971 to mid-1990s, and slightly narrowed in the years afterwards as manual workers and especially lower non-manual workers experienced faster gains in *M* than upper non-manual workers. In contrast, trends in *e*_65_- and *e*_75_ by occupational class show a widening in mortality inequalities among males over time, driven by slower increases in *e*_*x*_ among manual workers. Among females, differences across measures in the occupational class gradient were less pronounced. All measures showed a narrowing-widening-narrowing-widening inequality pattern over time, although the most recent widening was more pronounced for the *e*_*x*_ measures, mostly driven by stagnating mortality among manual workers.

Inequalities in *M* are larger on an absolute scale than inequalities in *e*_*x*_. This is to be expected, because as one compares average survival conditional on attaining older and older ages, there is less (remaining) room over which age at death can vary.

## Discussion

### Summary of results

Our study revealed that occupational disparities in *M* remained relatively stable until the 1990s and slightly declined in the years that followed. In contrast, the two *e*_*x*_ indicators showed an increase in disparities throughout the study period. The period proportion of participants that survived to *M* was stable throughout the near 50-year observation window at around 40% for all classes, and both sexes. The proportion surviving to age 65 or 75 differed by sex and occupational class, and increased substantially over time. Finally, all groups experienced mortality compression alongside increasing *M*. Compression was greater for the lower non-manual classes compared to the manual and upper non-manual classes, for both sexes. Towards the end of the follow-up period, *M* and *SD*(*M*+) for the lower non-manual class had nearly converged to those of the higher non-manual class.

### Interpretation

Our finding that all occupational classes were experiencing mortality compression at older ages is in contrast to the mortality dynamics observed over the entire age range in Finland and elsewhere. In Finland, variability in age at death for the manual classes increased since the 1970s, therefore suggesting an expansion rather than a compression of mortality, but declined for the non-manual classes. Similarly diverging trends over the entire adult age range have played out among income group in other Nordic countries [24], by educational groups in Spain [25], and the USA [26], and between quintiles of area-level deprivation in Scotland [27].

Moreover the pace of *M* increase and *SD*(*M*+) decline was comparable across occupational groups, being even slightly faster among the lower compared to upper occupational classes. Put together, these mortality dynamics suggest that once the lower occupational classes reach older ages they experience mortality improvements and mortality compression on par with the higher occupational classes even when inequalities are increasing over midlife. These results emphasize that mortality disparities depend on the age range over which inequalities are monitored and cannot be generalized over the entire age range.

How can we explain stable or narrowing inequalities in *M* and increasing inequalities by conditional life expectancies? Unlike *M*, *e*_*x*_ is conditional on the survival of individuals up to the threshold age x. Interpretation of trends in old-age mortality inequalities from period data with indicators such as these is challenging for several reasons. First, they assume that the characteristics of older individuals within successive cohorts remained stable over time and across social classes. However, today’s older people are not the same as yesterday’s. They live longer [28, 29], are in general healthier [30], have better cognition [31] and are better educated [32] than their peers from past generations. Therefore, the definition of old has changed over time. For instance, Finnish females in upper non-manual classes had an 80% chance of surviving to age 71 in 1971 and to age 81 in 2017 (calculations based on period life tables). More generally, over the past five decades the old-age survival front in high-income countries has shifted towards higher ages. Hence, the mortality level observed at age 68 today corresponds to age 65 a generation ago [33].

The characteristics of older individuals have also changed across socioeconomic groups. A survival-based definition of old, for instance, would not have the same meaning for the upper and lower social classes. Finnish females in manual classes had an 80% chance of surviving to age 68 in 1971 and 75 in 2017, which is about 3 and 6 years less than their counterparts in upper non-manual classes. Mortality differentials before the fixed age of truncation suggest that we are comparing groups that have experienced different degrees of mortality selection, and the secular declines in mortality mean that these have also changed over time. The top survivors of any cohort tend to be favorably selected on salubrious childhoods and health-promoting characteristics.

Second, indicators based on a fixed age threshold are capturing changing cause-of-death compositions which in part relate to changes in surviving past the age at truncation. The timing of these changes differs across social classes. In the 1970s, about 70% of cardiovascular deaths were occurring at ages 65+ with about half of these deaths occurring between ages 65–74 [34]. By 2017, the proportion of cardiovascular deaths at ages 65+ reached 90%, with the majority of these deaths occurring at ages 75+ [34]. The timing of these changes differs across social classes. In addition, the reduction of cardiovascular and cancer mortality has occurred at a different pace among the higher and lower classes. For example, the particularly rapid decline in smoking attributable mortality in the higher socioeconomic classes has led to significant changes in the cause-of-death distribution at older ages—changes that are being experienced more slowly in the lower socioeconomic classes. Thus, the substantive meaning of mortality inequalities beyond a fixed age are difficult to translate from one temporal-epidemiological setting to another, when the survival prospects and cause of death mixture have changed extensively over time and across social classes.

Third, improved survival from chronic conditions such as cardiovascular disease has led to a growing proportion of individuals who have experienced an illness at some point in their lifetime [35]. The rising number of survivors has been shown to slow the increase in life expectancy [36]. Survivors to major chronic diseases are more likely to die earlier than individuals who have never experienced an illness. In general, the disease prevalence of most common chronic diseases is higher among lower compared to higher educated groups [37]. Therefore, part of the reason for widening life expectancy disparities could come from a higher cluster of survivors with a past history of illness in lower than higher social classes. Inequalities in the modal age at death may be less affected because mortality declines only lead to increases in *M* when the declines occur at ages above *M* [9, 10]. To understand why, bear in mind that the death counts at any age are the product of the death rates and the survivors to that age. Reductions in death rates at only ages below *M* would increase the number of survivors to *M* but would not change the location of *M*. Reductions in death rates above *M* may increase *M*, but only when the higher number of survivors reaching an age above *M* compensates the lower death rates at that age [9].

On the other hand, there is no inherent reason why the two metrics should agree on the direction of inequality. Different conclusions are often drawn when measuring inequalities by standardized death rates on an absolute or relative scale [38, 39], by comparing the ratio of survivors to the ratio of deaths [40, 41] or by looking at inequalities in means versus inequalities in variation [42]. Each technique is valuable for exposing different dimensions of mortality inequalities. To be clear, we are not arguing that inequalities should never be measured from conditional age-at-death distributions, but rather that a multitude of alternative measures such as the modal age at death might bolster our interpretations of such trends. The modal age at death is particularly useful for monitoring inequalities as a marker of the most common age at death, and as an age beyond which a relatively stable proportion of adults survive.

Although our study focused on the Finnish case because of its long time series of mortality data by socioeconomic position, we would expect broadly similar patterns in other high-income countries. Compared to other European countries, Finland has moderately high levels of socioeconomic inequalities in mortality [38, 39, 43] although these are most pronounced over ages much younger than the mode. At older ages, these inequalities are more modest compared to other European countries [2–4].

### Limitations

Like all summary indicators of mortality, M has a number of mathematical properties which might limit the appropriateness of its use in all situations. First, it is insensitive to several transformations to the age at death distribution. As an example, if everyone who survived past the mode gained exactly one additional year of life, the location of the mode would be the same. However, since mortality generally changes smoothly over time, for most applications this should not be a major problem. Second, the mode is not a differentiable function of age-specific death rates [44, 45]. Therefore, assessing the contribution of changing age-specific death rates, causes of death, or group composition to changes in M is a challenge. Third, estimating M with great numerical precision inevitably involves some degree of smoothing which introduces uncertainty where the ages at death are grouped too coarsely. Much of the earlier demographic literature estimated *M* by fitting parametric models to the age-at-death distribution itself [13, 46]. This procedure was sensitive to the age range used and the underlying shape, or variability, of the distribution. By smoothing the hazard over a large age range instead, we reduce these biases.

However, achieved socioeconomic status is only attained in early adulthood, so some degree of left-truncation of the mortality data is necessary for studies of this type. We used data from the earliest age consistently available in our dataset, age 31, which is well below any estimates of *M*. We tested the sensitivity of our smoothing algorithm in selecting *M* by left-truncating our data at ages 35 and 40. These later starting ages, which are also well below *M*, resulted in the same estimate of *M*. Thus the smoothing procedure is not overly sensitive to the starting age, provided that it falls well below *M*. When left-truncation of the age domain is necessary, we recommend that users of this method always test the sensitivity of left-truncation of the age domain to capturing *M*, as we have done here.

We chose to smooth the hazard rate over age and period. Since less information is available for the first and last data points, trends need to be interpreted with some caution. In the most recent years, trends in *M* revealed a widening in occupational disparities between the upper non-manual and manual classes. To test the robustness of this result, we also estimated *M* from yearly and 5-year period age-at-death distributions smoothed over age only (one-dimensional smoothing). *M*-trends obtained from two-dimensional smoothed age-at-death distributions, our current approach, are similar to and exhibit less fluctuations than those estimated from one-dimensional distributions (S3 Fig). Therefore, the widening of occupational disparities in more recent years are not caused by our modeling procedure.

### Advantages of *M* in comparison to other approaches

The remarkably stable trend in the proportion of survivors to *M* is not solely specific to the Finnish national context. We made an additional analysis of the G7 countries, based on HMD data (HMD 2020) from 1970 to the latest year available, which also revealed stable trends in the proportion of the population surviving to *M*. This proportion oscillates between 0.30 (United States) and 0.43 (United Kingdom) for males and between 0.32 (United States) and 0.40 (Japan) for females (S2 Fig). *M* can therefore be viewed as the age at which a particular proportion of survivors is observed, in this case about 40%. Using a similar statistic (the number of life table deaths at or above the mode), [13] also found that this proportion has been stable in Japan since around 1960–64. As far as we are aware, this proportion has not been estimated in other settings.

Based on this feature, *M* is comparable to a characteristic-based measure of aging, a concept introduced by [47] for designating measures which reconceptualize age based on characteristics such as physical and cognitive abilities, health, or a number of survival-based characteristics. From the life table they proposed the age corresponding to a particular level of remaining life expectancy, to a particular level of mortality, or to a particular proportion of adult person-years lived. Such characteristics-based measures proposed by [47] have been used to examine past and future trends in population aging [30, 48]. These studies showed that when rescaling age by people’s characteristics the old age threshold increased from the traditional 65-year threshold.

To these measures we add *M*, which retains the advantages of percentile-based approaches in survivorship [33], with the additional attractivenesses that the percentage level is not chosen arbitrarily, is easily interpreted by the general population and is not sensitive to changes in the definition of premature mortality. We encourage the use of *M* for the analyses of levels and trends in socioeconomic differences in mortality; something that has not been carried out systematically before.

## Supporting information

S1 Fig Proportion of the population exposed to the risk of dying by occupational class and gender, Finland, 1971-2017. (TIF)

S2 Fig Proportion of survivors to *M* by sex, G7 countries since 1970. (TIF)

S3 Fig Modal age at death, *M*, estimated from one- and two-dimensional *P*-spline smooth age-at-death distributions, by occupational class and sex, Finland 1971-2017. (TIF)

S1 Appendix Estimation of occupation-specific modal age at death, *M*, and conditional life expectancies at age 65 and 75, *e*_65_, *e*_75_ from *P*-spline smooth occupation-specific density functions. (PDF)
