# Supplementary figures and images for: Why we should monitor disparities in old-age mortality with the modal age at death
Source: PLoS One. 2022 Feb 9;17(2):e0263626. doi: 10.1371/journal.pone.0263626 (PMC8827466; doi:10.1371/journal.pone.0263626)

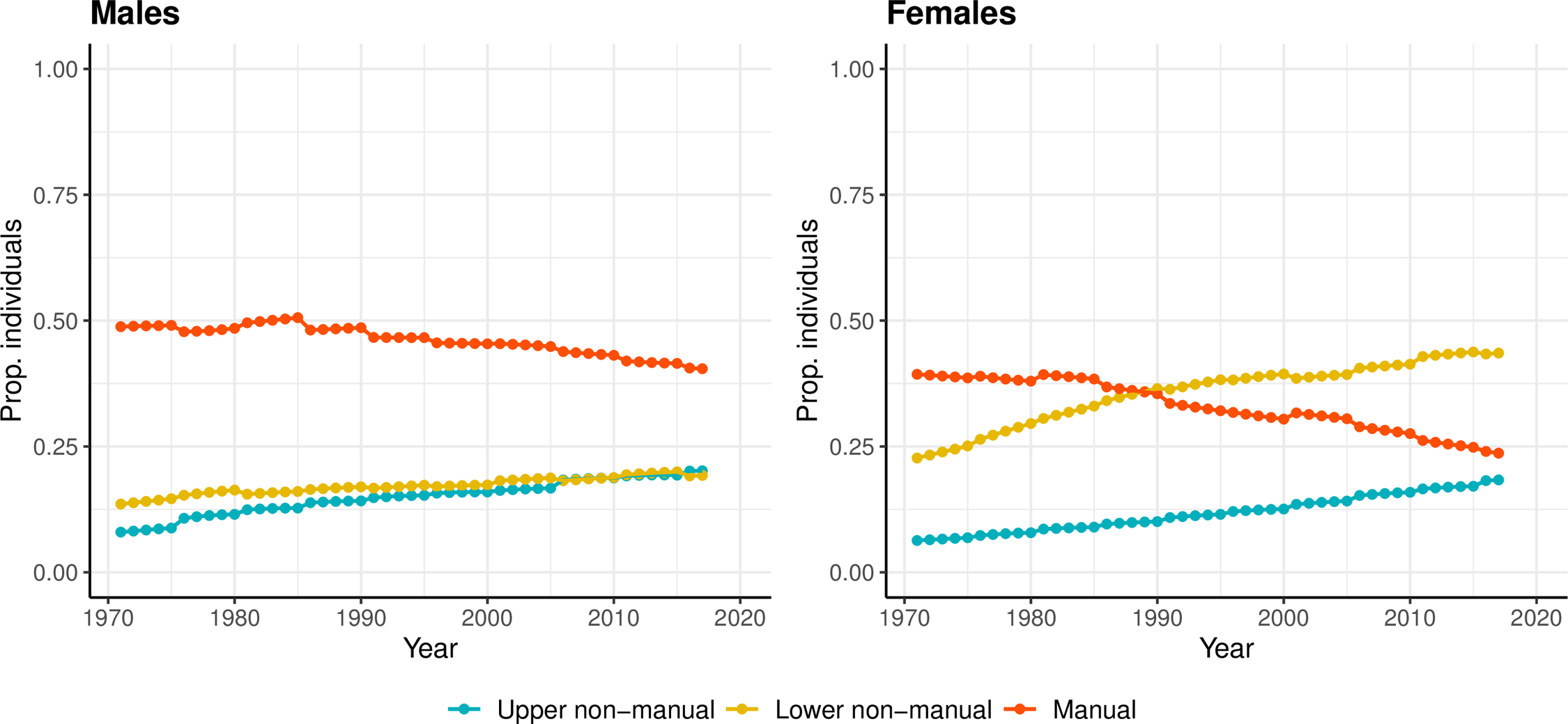

Supplement: S1 Fig — (TIF) [file pone.0263626.s001.tif]

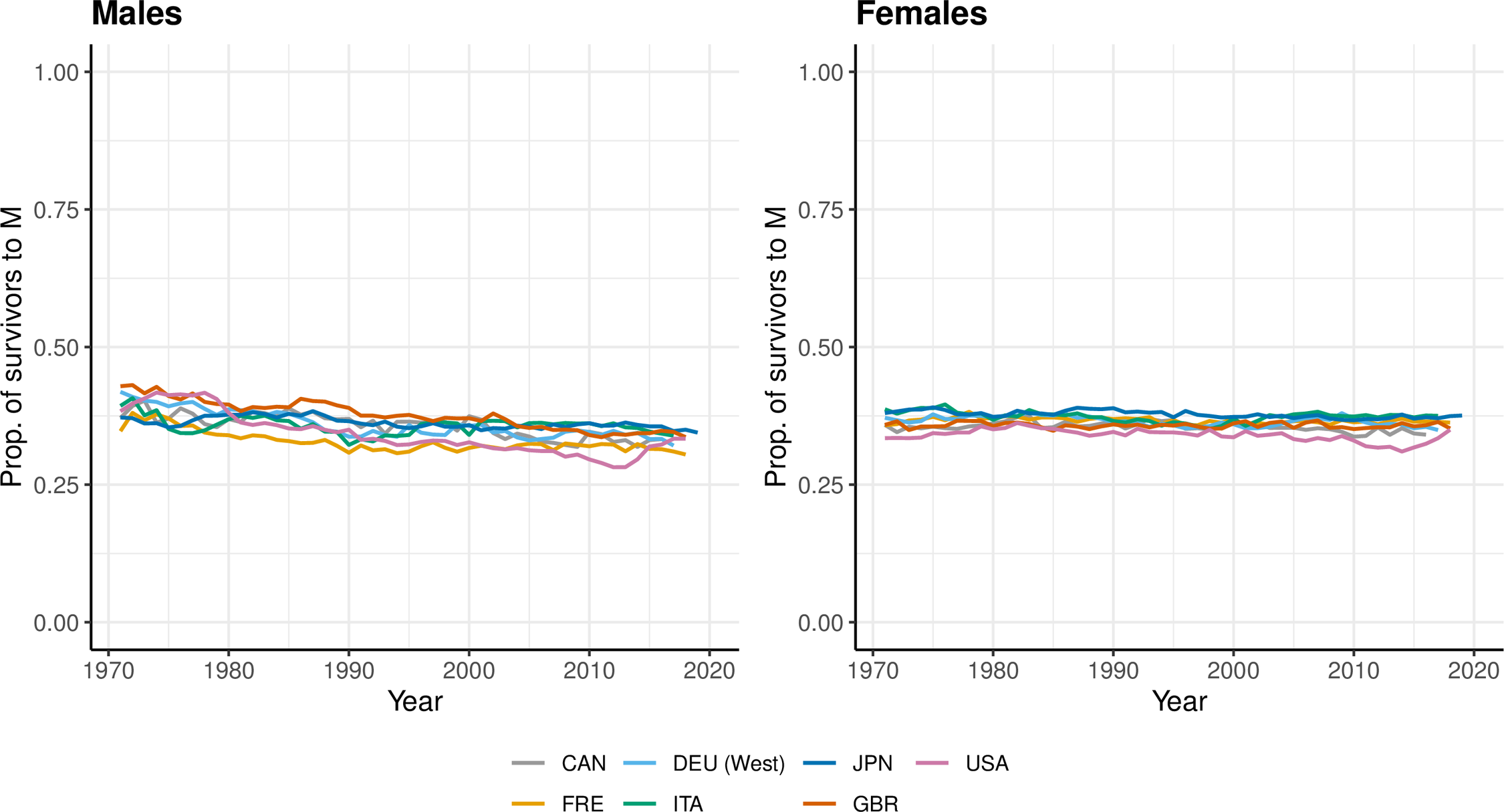

Supplement: S2 Fig — (TIF) [file pone.0263626.s002.tif]

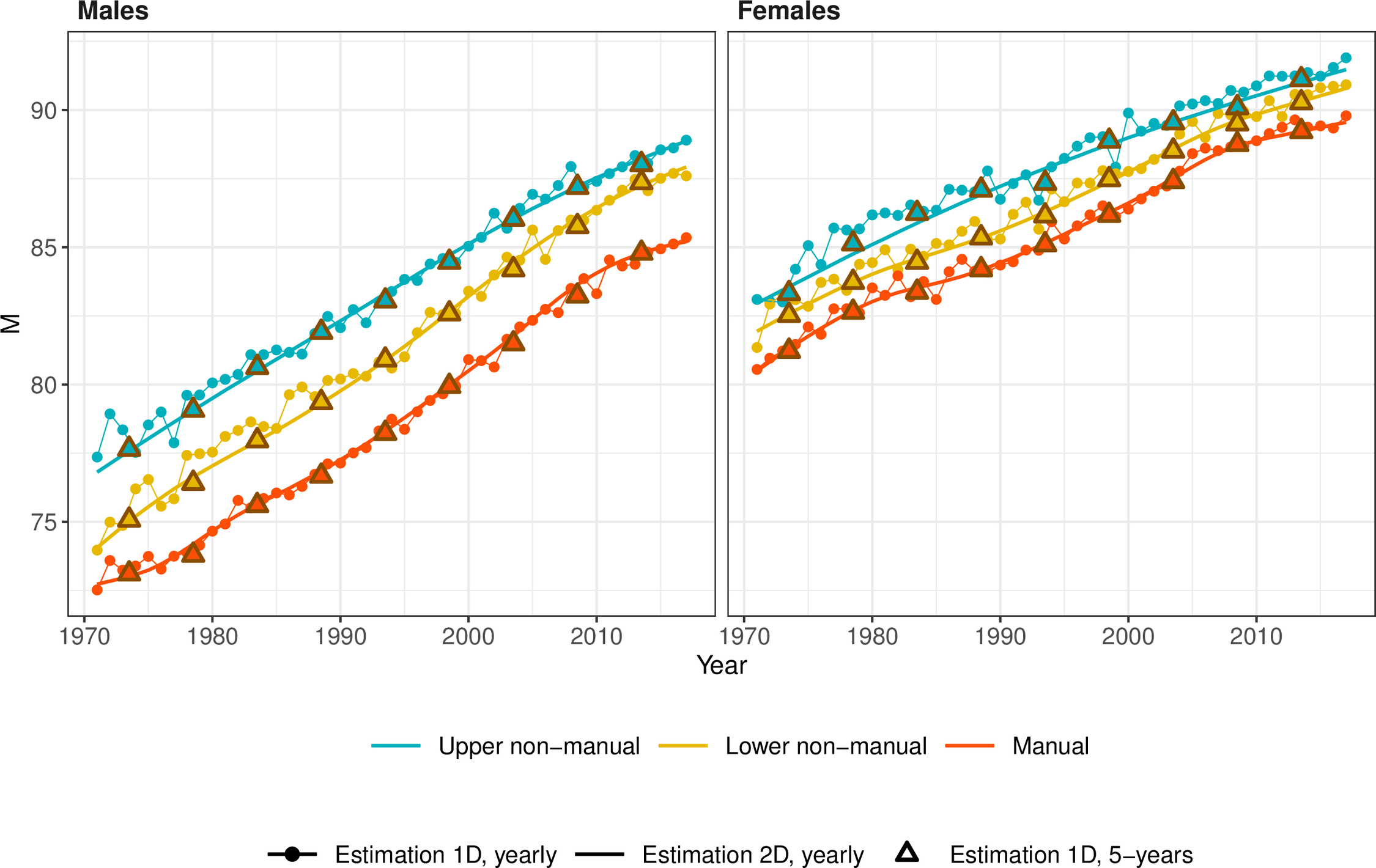

Supplement: S3 Fig — (TIF) [file pone.0263626.s003.tif]
