# Supplementary material for: Why we should monitor disparities in old-age mortality with the modal age at death
Source: PLoS One. 2022 Feb 9;17(2):e0263626. doi: 10.1371/journal.pone.0263626 (PMC8827466; doi:10.1371/journal.pone.0263626)
Supplement: S1 Appendix — (PDF) [file pone.0263626.s004.pdf]

S1 Appendix

For a given sex and occupational class, the density function $f(x, y)$, describing the age at death distribution, is continuous over age x and calendar year y. It is obtained by multiplying the force of mortality $\mu(x, y)$ and survival function, $S(x, y)$, such as:

$$f(x,y) = \mu(x,y)S(x,y) = \mu(x,y)exp(-\int_0^x \mu(u,y)du) \quad (1)$$

The survival function can therefore be solely expressed in terms of the force of mortality as shown in the last term in Eq A.1.

Thus, the computation of sex- year- and occupation-specific modal age at death, M, and conditional life expectancies, e_x, depend solely on the estimation of the corresponding forces of mortality, as:

$$M(y) = \max_x \ f(x,y) = \max_x \mu(x,y)exp\Big(-\int_0^x \mu(u,y)du\Big) \quad (2)$$

$${}_x(y) = \frac{\int_x^\omega S(a,y)da}{S(x)} = \int_x^\omega exp\Big(-\int_x^a \mu(u,y)du\Big)da. \quad (3)$$
